# Cytotoxicity of cytokine-induced killer cells targeted by a bispecific antibody to gastric cancer cells

**DOI:** 10.3892/ol.2013.1281

**Published:** 2013-04-02

**Authors:** LIN ZHANG, YANHONG HOU, JIAN ZHANG, JING HU, KUNPENG ZHANG

**Affiliations:** 1 Department of Gastroenterology, The 309 Hospital of the PLA, Beijing 100091;; 2 Department of Molecular Biology, The Fourth Military Medical University, Xi’an 710032, P.R. China

**Keywords:** gastric cancer, cytokine-induced killer cell, bispecific antibody, immunotherapy

## Abstract

The aim of the present study was to investigate the cytotoxic activity of cytokine-induced killer (CIK) cells targeted by an epidermal growth factor receptor (EGFR)/CD3 bispecific antibody (BsAb) to the gastric cancer cell line SGC7901. A BsAb was constructed by chemically cross-linking a monoclonal antibody (McAb) against human CD3 with another McAb against human EGFR. An immunocytochemistry assay was performed to detect the expression of EGFR in SGC7901 cells. The cytotoxic activity of CIK cells targeted by the EGFR/CD3 BsAb was analyzed by the ^51^Cr release assay, Subsequently, a comparison of the cytotoxic activity between CIK cells targeted by EGFR/CD3 BsAb, CIK cells targeted by EGFR McAb or/and CD3 McAb and CIK cells was performed. The antineoplastic activity of the CIK cells directed using the EGFR/CD3 BsAb *in vivo* was analyzed by tumor growth and tumor reduction assays. The cell lysis rate of CIK cells targeted by the EGFR/CD3 BsAb was higher compared with those of CIK cells targeted by CD3 McAb only or by CD3 McAb and EGFR McAb. The lysis rates of the latter two groups were significantly higher than those of CIK cells targeted by EGFR McAb only and CIK cells (P<0.05). The mean tumor reduction using the administration of CIK cells directed by the EGFR/CD3 BsAb was higher than those of the other groups (P<0.05). The results indicate that the EGFR/CD3 BsAb is able to enhance the ability of CIK cells to bind to and kill gastric cancer cells *in vitro* and *in vivo*.

## Introduction

At present, gastric cancer is an extremely common malignancy. Although the incidence of the disease has been declining for the past few decades, each year ∼798,000 people are diagnosed with gastric cancer worldwide (9.9% of total cancer cases) and 628,000 people succumb to the disease (12.1% of cancer mortalities) (1). Globally, the majority of cases of gastric cancer have been in East Asia, in countries such as China, Japan and Korea. In China, the overall prognosis for this cancer remains poor since the majority of patients are diagnosed with advanced disease due to the lack of an effective screening method or strategy. Consequently, the 1- and 5-year survival rates of patients suffering from gastric cancer are extremely low (2). Although adjuvant treatments, including chemotherapy and radiotherapy are frequently used, their overall impact on the prognoses of these patients remains limited and their adverse reactions frequently affect the patients’ quality of life. A new therapeutic strategy may be an urgent requirement for gastric cancer therapy.

Cellular immunotherapy against solid tumors has become a promising treatment following a number of preclinical and clinical trials. Of the numerous effector cells, cytokine-induced killer (CIK) cells have received considerable attention. CIK cells are expanded *in vitro* from peripheral blood mononuclear cells (PBMCs) by the addition of interferon-γ (IFN-γ), interleukin (IL)-2, IL-1 and a monoclonal antibody (McAb) against CD3 (3,4). CIK cells are highly efficient cytotoxic effector cells with the co-expression of CD3 and CD56 and NK activity (5). CIK cells have been used as effector cells in the adoptive cell therapy against certain cancers and have shown a promising effect (6,7).

Bispecific antibodies (BsAbs) are antibodies which are made by chemical or biological methods and not observed in natural conditions. They are typically designed to recognize a specific epitope on effector cells and a target epitope on tumor cells simultaneously. In comparison with conventional mAbs, BsAbs are able to link immune effector cells to tumor cells directly and have a more powerful ability to activate the immune-mediated destruction of cancer cells. In 1994, Beun *et al*(8) reported that BsAbs directed to a target-cell surface antigen and the T-cell-receptor (TCR)/CD3-complex mediated the activation of T cells and the induction of target-cell lysis by the activated cells. Therapeutic strategies using BsAbs have been performed in a number of basal and preclinical studies (9–13). However, a therapeutic strategy using a combination of CIK cells and BsAbs against gastric cancer has not yet been reported. The present study investigated the cytotoxic activity of CIK cells targeted by epidermal growth factor receptor (EGFR)/CD3 BsAbs against the gastric cancer cell line SGC7901 to analyze the effect of this therapeutic strategy against gastric cancer.

## Materials and methods

### Materials

EGFR McAb was purchased from Beijing Zhongshan Biotechnology Co., Ltd. (Beijing, China). Rabbit anti-mouse HPR (1:1,000; Dako, Copenhagen, Denmark) was used to recognize the corresponding proteins. Common cell culture plates were purchased from Orange Scientific (Braine-l’Alleud, Belgium). EGFR, CD3 and CD56 McAbs were purchased from Biosynthesis Co., Ltd. (Beijing, China). A total of 50 8–10-week-old female nude mice weighing 18–22 g (NU/NU; Iffa Credo, l’Arbresle, France) were purchased from Biotechnology Co., Ltd. (Beijing, China) and housed in self-contained filter-top cages (5 mice/cage). Approval of the human blood collection protocol was obtained from The Beijing Blood Center the study was approved by the Experimental Animal Investigation Committee of The 309 Hospital of PLA, Beijing, China.

### Cell lines and culture

The human gastric adenocarcinoma cell line (SGC7901) was obtained from the Shanghai Cell Research Institute of the Chinese Scientific Academy (Shanghai, China) and stored and transfer cultured in The Laboratory of the Gastroenterology Department at The 309 Hospital. DMEM containing 10% calf serum, 100 IU/ml penicillin and 100 IU/ml streptomycin was used as a conventional culture medium. The culture procedures were performed at 37°C, 5% CO_2_ and saturation humidity.

### Investigation of EGFR expression in the SGC7901 cell line

The expression of EGFR in SGC7901 cells was detected to determine whether the cell lines were suitable for use in the study. An immunocytochemical assay and RT-PCR were performed and the results showed that EGFR was expressed in the cell line.

### Production of crosslinked anti-CD3 and anti-EGFR BsAb

The anti-CD3 × anti-EGFR (EGFR/CD3) BsAb was produced using the chemical heteroconjugation technique described previously by Sen *et al*(14). Anti-CD3 McAb (1 mg/ml) in 50 mM NaCl and 1 mM EDTA at pH 8.0 was added to a 5-fold molar excess of Traut’s reagent (2-iminothiolane HCl; Pierce, Rockford, IL, USA) at room temperature for 1 h. Anti-EGFR (1 mg/ml) in 0.1 mol/l sodium phosphate and 0.15 mol/l NaCl (pH 7.2) were then added to a 4-fold excess of sulfosuccinimidyl-4-(N-maleimidomethyl) cyclohexane-1-carboxylate (Pierce) at room temperature for 1 h. The two antibodies were purified on PD-10 columns (Pharmacia, Uppsala, Sweden) in PBS to remove the unbound cross-linker. The cross-linked McAbs were mixed immediately in equimolar ratios and conjugated at 4°C overnight. The product was analyzed using SDS-PAGE (2–15% gradient; OWL Scientific, Woburn, MA, USA) and detected using Coomassie blue staining.

### Preparation of CIK cells

The CIK cells were prepared according to the method in the study by Tita-Nwa *et al*(15). PBMCs were isolated from healthy donors via the blood bank of Beijing. PBMCs were isolated by Ficoll density gradient centrifuging, washed with RPMI-1640 and then resuspended in RPMI-1640 containing 10% calf serum, 100 IU/ml penicillin, 100 IU/ml streptomycin and INF-γ (500 U/ml). On day 1, IL-2 (400 U/ml), IL-1α (100 U/ml) and anti-CD3 McAb (20 ng/ml) were added. The culture media were changed every 2 days and the concentration of the cells was regulated to 2×10^6^/ml. The cells were cultured for 21 days and detected using a flow cytometry assay.

### Binding of EGFR/CD3 BsAb to effector cells and target cells

The binding of the EGFR/CD3 BsAb to SGC7901 cells and CIK cells was detected by indirect immunofluorescence methods. The binding of the EGFR/CD3 BsAb to SGC7901 cells was detected using FITC-labeled CD3 antigen, while the binding of the EGFR/CD3 BsAb to CIK cells was detected with FITC-labeled EGFR antigen. The binding rates were detected using a flow cytometry assay.

### Cytotoxicity assays

^51^Cr release assays were performed to detect the cytotoxicity of CIK cells to gastric cancer cells targeted by EGFR/CD3 BsAb as described previously by Hoyle *et al*(16) and Chan *et al*(17). SGC7901 cells (1×10^6^) were labeled with 300 *μ*Ci sodium chromate (Dupont-NEM, Boston, MA, USA). The labeled cells were then washed twice with PBS, suspended in RPMI-1640 and plated in 96-well plates at 1×10^4^ cells per well, in triplicate. Effector cells and antibodies were added to form groups as follows: group A: EGFR/CD3 BsAb (20 *μ*g/ml) + CIK cells (5×10^6^/ml) + SGC7901 cells (1×10^5^/ml); group B: CD3 McAb (20 *μ*g/ml) + CIK cells (5×10^6^/ml) + SGC7901 cells (1×10^5^/ml); group C: EGFR McAb (20 *μ*g/ml) + CIK cells (5×10^6^/ml) + SGC7901 cells (1×10^5^/ml); group D: CD3 McAb (20 *μ*g/ml) + EGFR McAb (20 *μ*g/ml) + CIK cells (5×10^6^/ml) + SGC7901 cells (1×10^5^/ml); and group E: CIK cells (5×10^6^/ml) + SGC7901 cells (1×10^5^/ml). CIK cells were added as effector cells at effector:target (E:T) cell ratios of 50:1 and incubated at 37°C, 5% CO_2_ for 4 h. The radioactivity of the supernatant was measured in a γ counter (Cobra/AII; Packard BioScience, Meriden, CT, USA). The lysis rate was calculated according to the following formula: Lysis rate (%) = [(sample release) − (spontaneous release) / (maximum release) − (spontaneous release)] × 100.

### Cytotoxicity assays at various E:T cell ratios

The cytotoxicity of CIK cells targeted by the EGFR/CD3 BsAb to gastric cancer cells was analyzed by ^51^Cr release assays at various E:T cell ratios. The assays were performed as described previously. CIK cells were added as effector cells at E:T cell ratios (1:1, 10:1, 20:1, 30:1, 40:1, 50:1, 60:1,70:1, 80:1, 90:1 and 100:1) and incubated at 37°C, 5% CO_2_ for 4 h, then the lysis rates of the groups were measured. The results reported are the mean values of three independent experiments performed in triplicate and a curve was established based on the mean lysis rates.

### Cytotoxicity analysis in vivo

The cytotoxicity of CIK cells targeted by the EGFR/CD3 BsAb to gastric cancer cells was investigated by therapeutic experiments in a xenograft mouse model. Each mouse was injected s.c. in the right flank with 5×10^6^ SGC7901 cells in 0.2 ml PBS. After 10 days, the mice with tumors (diameter ≥0.5 cm) were selected for the therapeutic experiment. The selected mice were assigned to 8 treatment groups (5 mice per group), as follows: group A: EGFR/CD3 BsAb (1 mg) + CIK cells (1×10^9^/ml); group B: CD3 McAb (1 mg) + CIK cells (1×10^9^/ml); group C: EGFR McAb (1 mg) + CIK cells (1×10^9^/ml); group D: CD3 McAb (1 mg) + EGFR McAb (1 mg) + CIK cells (1×10^9^/ml); group E: CD3 McAb (1 mg); group F: EGFR McAb (1 mg) group G: EGFR/CD3 BsAb (1 mg); and group H: 0.9% NaCl i.v. injection alone (0.2 ml/injection). All antibodies and effector cells were injected i.v. into the heat-dilated tail vein and the first day of treatment was day 0. The treatments were performed with twice weekly measurements of tumor dimensions and estimation of tumor volumes (mm^3^) using the formula: V = a × b^2^/2, where a is the length and b is the width. A tumor growth curve of each group was established based on the mean tumor volume at various times. At day 35, the tumors were dissected and weighed, then the tumor inhibition rates were calculated by the following formula: Tumor inhibition rate (%) = [(mean tumor weight of group G) − (mean tumor weight of each experimental group) / (mean tumor weight of group G)] × 100.

### Investigation of EGFR and CD3 expression in tumor tissues following treatment

The expression levels of EGFR and CD3 in the tumor tissues of the xenograft mice following treatment were detected to determine whether the effector cells were able to migrate into the tumor tissues. The tumors were dissected and embedded in paraffin. Tissue sections of 5-*μ*m thickness were cut from paraffin-embedded tissue blocks, then H&E staining and immunohistochemical assays were performed.

### Statistical analysis

In present study, the data are expressed as mean ± SD. One-way ANOVA test and the Student’s t-test were performed. All data were analyzed with the SPSS 11.0 (SPSS Inc., Chicago, IL, USA) statistical software package. P<0.05 was considered to indicate a statistically significant difference.

## Results

### Expression of EGFR in SGC7901 cells

**Figure 1:**
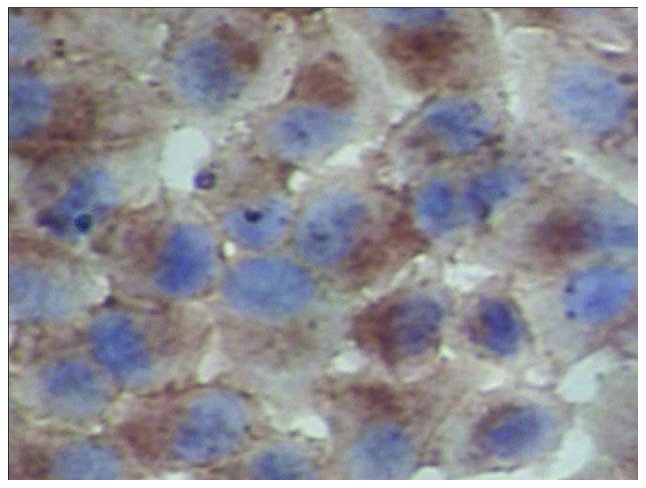
Immunostaining of EGFR in SGC7901 cells. The brown positive signals were mainly distributed in the cell membranes and cytoplasm and there was no clear positive signal in the cell nucleus. EGFR, epidermal growth factor receptor.

**Figure 2:**
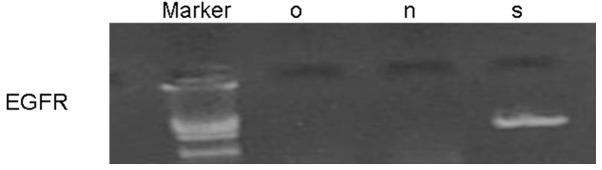
RT-PCR assay for EGFR expression in SGC7901 cells. The results showed that there was EGFR expression in the SGC7901 cell line at the mRNA level. EGFR, epidermal growth factor receptor.

The expression of EGFR in the gastric adenocarcinoma cell strain SGC7901 was detected by RT-PCR and immunocytochemical analysis. The results in the SGC7901 cells were positive. The immunohistochemical assay showed that the expression of EGFR in SGC7901 cells was mainly distributed in the cell membrane and cytoplasm and there was no clear positive signal in the cell nucleus (Figs. 1 and 2).

### Investigation and characterization of CIK cells

**Figure 3:**
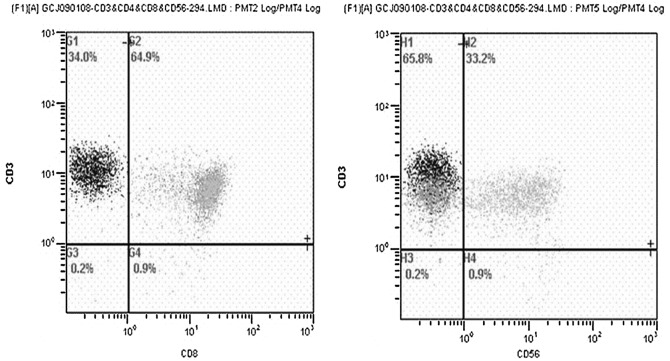
Flow cytometry assay of CIK cells. The result showed that the populations of CD3^+^CD8^+^ T cells accounted for ∼64.9%, while CD3^+^CD56^+^ T cells accounted for ∼33.2%. CIK, cytokine-induced killer.

The populations of cells in expanded CIK cells were analyzed using flow cytometric analysis at day 21, to evaluate the cytotoxicity of the CIK cells. The results suggested that populations of CD3^+^CD8^+^ T cells accounted for ∼64.9% and CD3^+^CD56^+^ T cells accounted for ∼33.2% (Fig. 3).

### Binding of EGFR/CD3 BsAb

**Figure 4:**
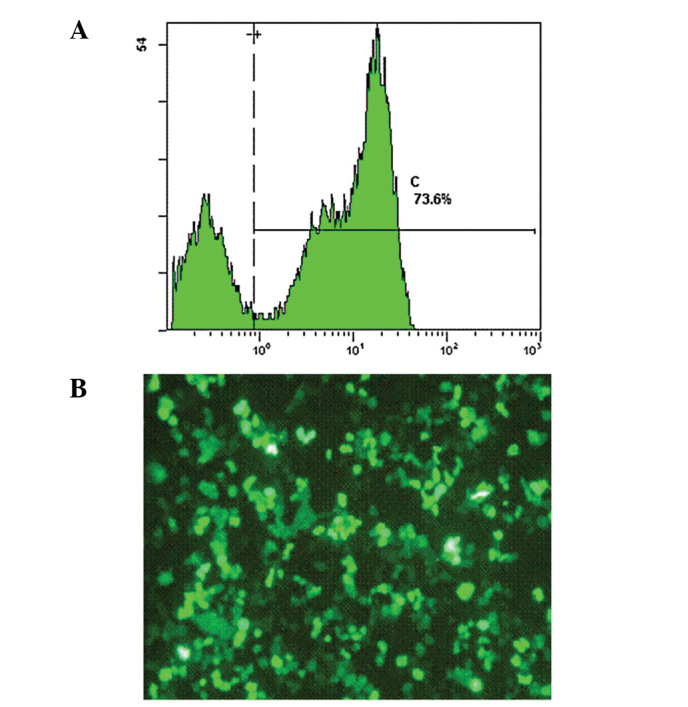
(A) Binding of EGFR/CD3 BsAb to target cells analyzed by flow cytometry and (B) under fluorescene microscope. (FITC staining; magnification, ×100). The results showed that the binding rate of EGFR/CD3 BsAb to SGC7901 cells was 73.6%. EGFR, epidermal growth factor receptor; BsAb, bispecific antibody.

The binding activity of the EGFR/CD3 BsAb to SGC7901 and CIK cells was studied using an indirect immunofluorescence method and the binding rates were analyzed with a flow cytometry assay. The results showed that the EGFR/CD3 BsAb had a high binding activity with regard to CIK and SGC7901 cells, which revealed the dual affinity of the BsAb for the target and effector cells. The binding rates of the EGFR/CD3 BsAb to SGC7901 and CIK cells were 73.6 and 71.2%, respectively (Figs. 4 and. 5).

### Cytotoxicity at various E:T cell ratios

**Figure 6:**
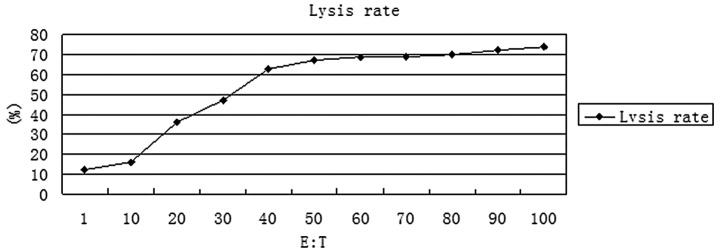
Cytotoxicity at various E:T cell ratios. CIK cells targeted by EGFR/CD3 BsAb exhibited considerable cytotoxicity at E:T cell ratios of ∼50:1. E:T, effector:target; CIK, cytokine-induced killer; EGFR, epidermal growth factor receptor; BsAb, bispecific antibody.

The cytotoxicity of CIK cells targeted by the EGFR/CD3 BsAb at various E:T cell ratios was tested by ^51^Cr release assays. The results of the assays are shown in Fig. 6. At an E:T cell ratio of ∼50:1, CIK cells targeted by the EGFR/CD3 BsAb exhibited considerable cytotoxicity. The cytotoxicity of CIK cells did not increase significantly when the E:T cell ratio increased beyond 50:1.

### Enhancement of CIK cell cytotoxicity by EGFR/CD3 BsAb

**Figure 7:**
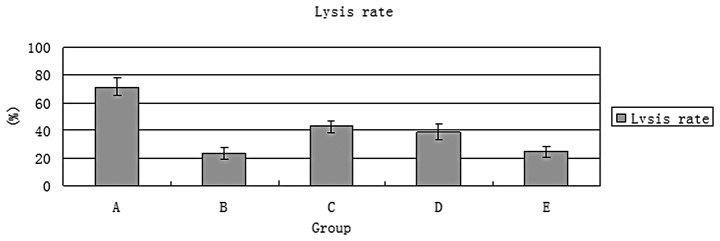
^51^Cr release assays. At an E:T cell ratio of 50:1, CIK cells directed by EGFR/CD3 BsAb had a cell lysis rate of 71.4% which was higher than those of the other groups. E:T, effector:target; CIK, cytokine-induced killer; EGFR, epidermal growth factor receptor; BsAb, bispecific antibody; McAb, monoclonal antibody; A, EGFR/CD3 BsAb (20 *μ*g/ml) + CIK cells (5×10^6^/ml) + SGC7901 cells (1×10^5^/ml); B, CD3 McAb (20 *μ*g/ml) + CIK cells (5×10^6^/ml) + SGC7901 cells (1×10^5^/ml); C, EGFR McAb (20 *μ*g/ml) + CIK cells (5×10^6^/ml) + SGC7901 cells (1×10^5^/ml); D, CD3 McAb (20 *μ*g/ml) + EGFR McAb (20 *μ*g/ml) + CIK cells (5×10^6^/ml) + SGC7901 cells (1×10^5^/ml); E, CIK cells (5×10^6^/ml) + SGC7901 cells (1×10^5^/ml) .

The cytotoxicity of CIK cells directed by the EGFR/CD3 BsAb was tested by a cell lysis assay and compared with that of CIK cells directed by EGFR or CD3 McAbs. The results of the assay are shown in Fig. 7. At an E:T cell ratio of 50:1, CIK cells directed by EGFR/CD3 BsAb exhibited a cell lysis rate of 71.4%, which was higher than those of CIK cells directed by EGFR or CD3 McAbs and the other control groups (P<0.05).

### Effect of CIK cells directed by EGFR/CD3 BsAb on tumor growth

**Figure 8:**
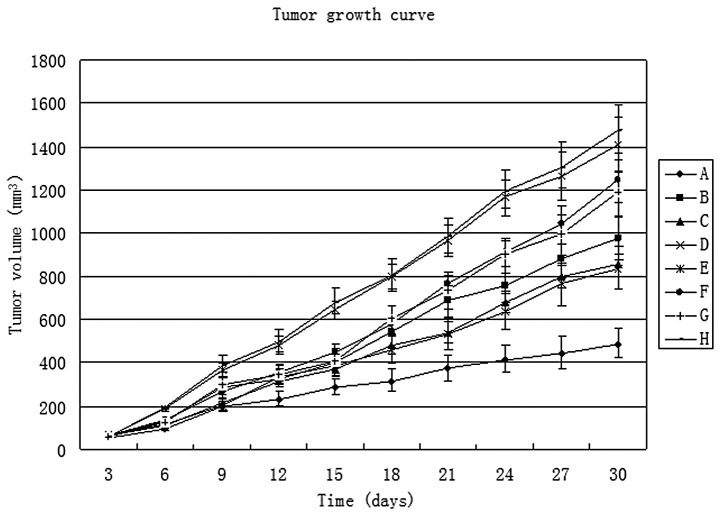
Tumor growth assay. The assay showed that the tumors in mice treated with CIK cells directed by the EGFR/CD3 BsAb grew significantly slower than those in the other groups (P<0.05). EGFR, epidermal growth factor receptor; CIK, cytokine-induced killer; BsAb, bispecific antibody; McAb, monoclonal antibody; A, EGFR/CD3 BsAb (1 mg) + CIK cells (1×10^9^/ml); B, CD3 McAb (1 mg) + CIK cells (1×10^9^/ml); C, EGFR McAb (1 mg) + CIK cells (1×10^9^ 1ml); D, CD3 McAb (1 mg) + EGFR McAb (1 mg) + CIK cells (1×10^9^/ml); E, CD3 McAb (1 mg); F, EGFR McAb (1 mg); G, EGFR/CD3 BsAb (1 mg); H: 0.9% NaCl i.v. injection alone (0.2 ml/injection).

The antineoplastic activity of CIK cells directed by the EGFR/CD3 BsAb was analyzed with tumor growth and tumor reduction assays. The results are shown in Table I and Fig. 8. The EGFR/CD3 BsAb-redirected CIK cells showed a significant enhancement of antitumor activity in the mice. The results of the tumor growth curve assay showed that the tumors in mice treated with CIK cells directed by the EGFR/CD3 BsAb grew significantly slower than those in the other groups (P<0.05; Fig. 8). The administration of CIK cells directed by the EGFR/CD3 BsAb caused a mean tumor reduction of 69.8%, which was higher than those of other groups (P<0.05; Table I). The administration of CD3 McAb alone without CIK cells had no significant antineoplastic effect compared with that in the control group treated with 0.9% NaCl. CD3 McAb did not enhance the cytotoxicity of CIK cells.

### Expression of EGFR and CD3 in tumor tissues following treatment

**Figure 9:**
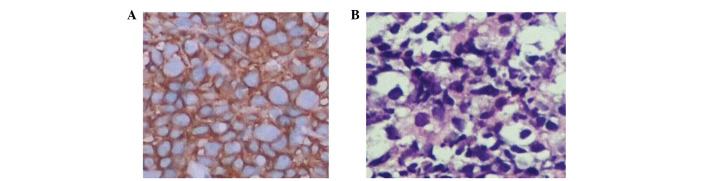
Immunostaining of EGFR in tumor tissues following treatment. (A) Immunohistochemical analysis of EGFR. Brown positive signals were observed in tumor tissues of all groups and were distributed in the cytoplasm and membrane. (B) H&E staining of tumor tissues. EGFR, epidermal growth factor receptor.

**Figures 10.**
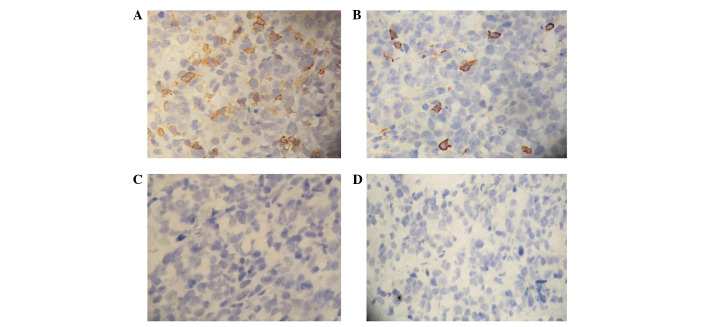
Immunostaining of CD3 in tumor tissues following treatment. (A) Immunohistochemical analysis of CD3 in group A. Brown positive signals were distributed in the cytoplasm and membrane and the positive cells were significantly more numerous than those of the other groups. (B) Immunohistochemical analysis of CD3 in group C. There were a few positive cells which were distributed in the tumor tissue but significantly fewer than in group A. (C) Immunohistochemical analysis of CD3 in group B. There was no clear positive signal in the tumor tissues of group B. (D) Immunohistochemical analysis of CD3 in group H. There was no clear positive signal in the tumor tissues of group H. Group A, EGFR/CD3 BsAb (1 mg) + CIK cells (1×10^9^/ml); group B, CD3 McAb (1 mg) + CIK cells (1×10^9^/ml); group C, EGFR McAb (1 mg) + CIK cells (1×10^9^ 1ml); group H: 0.9% NaCl i.v. injection (0.2 ml/injection). EGFR, epidermal growth factor receptor; CIK, cytokine-induced killer; BsAb, bispecific antibody; McAb, monoclonal antibody.

The immunohistochemical analysis revealed marked positive expression of EGFR in the tumor tissues of all groups and the positive signals were distributed in the cytoplasm and membrane (Fig. 9). The immunohistochemical assay showed that the expression of CD3 in the tumor tissues of the mice treated with the EGFR/CD3 BsAb-redirected CIK cells was increased significantly and the positive signals were mainly distributed in the membrane and cytoplasm (Fig. 10). No positive signals were observed in the tumor tissues of the mice treated with antibodies alone without CIK cells. These results indicate that the EGFR/CD3 BsAb was able to direct CIK cells to tumor tissues and enhanced their cytotoxicity to gastric cancer cells *in vivo*.

## Discussion

Although the morbidity and mortality of gastric cancer has been reduced, gastric cancer remains a common disease in East Asia, with a poor prognosis and low survival rates. In China, the prognosis of gastric cancer is poor since mass screening and early diagnosis are not feasible. The therapeutic effects of traditional strategies are not satisfactory as the pathogenesis and molecular control of gastric cancer are poorly understood, although the involvement of a number of affecting factors has been proposed. Among these factors, cellular immune defects are common in patients with gastric adenocarcinoma, as well as other malignancies, which may have diverse effects in regulating critical antitumor functions, including immunological recognition and regulation and cytotoxicity. Therefore, cellular immunotherapy has been proposed to be an important adjunctive therapy for gastric cancer and has yielded promising effects. As the most common type of effector cell, CIK cells express the T cell marker CD3 and NK cell marker CD56 and develop cytotoxic activity against various cancer cells, including those of gastric cancer. To enhance cytotoxity and antitumor specificity, certain artificial BsAbs have been used to direct CIK cells to tumor cells in previous studies (18–20).

The EGFR/CD3 BsAb directs CIK cells to tumor cells by connecting CD3 on effector cells with EGFR on tumor cells. It has been demonstrated that CD3 is an important triggering molecule that activates T cells (21–24). However, EGFR McAb was able to block the EGFR and restrain tumor cell growth in a number of preclinical and clinical studies (25,26). Theoretically, the EGFR/CD3 BsAb binds to EGFR and CD3 simultaneously, so it should not only activate T cells/CIK cells, but also restrict tumor cell growth. In the present study, a EGFR/CD3 BsAb was produced and the cytotoxicity of CIK cells targeted by this BsAb to gastric cancer cells was investigated.

The results of cytotoxicity assays suggested that redirected lysis was increased significantly when CIK cells were combined with the EGFR/CD3 BsAb. CD3 McAb did not enhance tumor cell lysis compared with the other groups. It was observed in the present study that cytotoxicity increased to some extent when CIK cells were combined with EGFR McAb. With regard to the expression of Fc receptors (CD16, CD32 or CD64) on CIK cells, EGFR McAb also directed effector cells to EGFR positive target cells by antibody dependent cellular cytotoxicity (ADCC). However, the ability of EGFR McAb to enhance cytotoxicity was more limited compared with that of the EGFR/CD3 BsAb. Tita-Nwa *et al* demonstrated that CD19/CD5 bsAb enhanced the cytotoxicity of CIK cells against CD19^+^ B cell lymphoma lines *in vitro* and suggested that these results supported the experimental use of the *in vivo* bsAb models (15). Chan *et al* investigated the cell killing ability of CIK cells against primary ovarian carcinoma cells with and without BsAbs and revealed that a CA125/Her2 BsAb significantly enhanced the cytotoxicity of CIK cells in primary ovarian cancer cells in a mouse model (17). These results provide more support for the role of BsAbs in enhancing the cytotoxicity of CIK cells. No studies on this therapeutic strategy for gastric cancer have been reported previously, so the present study is the first to report that a EGFR/CD3 BsAb is able to enhance the cytotoxicity of CIK cells to gastric cancer cells *in vitro* and *in vivo*.

The results of the cytotoxicity assays *in vivo* showed that the antitumor activity of CIK cells was enhanced significantly by the EGFR/CD3 BsAb when compared with EGFR or CD3 McAbs in a nude mouse model. The present results are in agreement with those of other studies which used different BsAbs to target T cells or CIK cells to malignant cells (27,28). These findings have demonstrated the promising therapeutic effect and clinical potential of this strategy in a number of malignant tumors. Certain researchers (29) have used bioluminescent imaging to serially observe the response to therapy without the need for sacrificing the experimental animals. We were unable to perform bioluminescent imaging due to a lack of equipment, but we considered that it was possible to obtain credible data using the present test methods.

The immunohistochemical assays revealed that EGFR expression was common in tumor tissues and the positive signals were distributed in the cell membrane and cytoplasm. EGFR is the most important member of the EGFR family. The overexpression of EGFR occurs in numerous human malignancies, including lung, breast, colon and gastric carcinomas (30–32). It is well known that EGFR is associated with malignant transformation and tumorigenesis, so this molecule has been regarded as an important target in numerous preclinical and clinical studies. The overexpression of EGFR in gastric cancer has been confirmed in a number of studies (33,34). Certain EGFR McAbs have been generated and used against a variety of malignancies. One such antibody, cetuximab, has yielded a promising therapeutic effect in delaying gastric cancer progression (35,36). Consequently, EGFR was selected as the target in gastric cancer cells in the present study.

The present results showed that the positive immunohistochemical staining of CD3 was distributed in the cell membrane and cytoplasm of certain cells in the tumor tissues of groups A, C and D. However, there were no clear positive signals in the tumor tissues of the other groups. The CD3 positive cells in the mice treated with EGFR/CD3 BsAb-redirected CIK cells were significantly more abundant than those in the mice treated with EGFR McAb-redirected CIK cells. The results suggested that the EGFR/CD3 BsAb and EGFR McAb were able to direct CD3 positive CIK cells to tumor cells and the ability of the EGFR/CD3 BsAb to target CIK cells to tumor cells was higher than that of EGFR McAb.

Given the unfavorable prognosis of patients with advanced gastric cancer, there is a marked impetus to investigate new therapeutic strategies to improve the outcome for these patients. The results of the present investigation demonstrated that the EGFR/CD3 BsAb significantly enhanced the cytotoxic activity of CIK cells to gastric cancer cells *in vitro* and *in vivo*, so this BsAb may potentially aid cellular therapy.

## Figures and Tables

**Figure 5:**
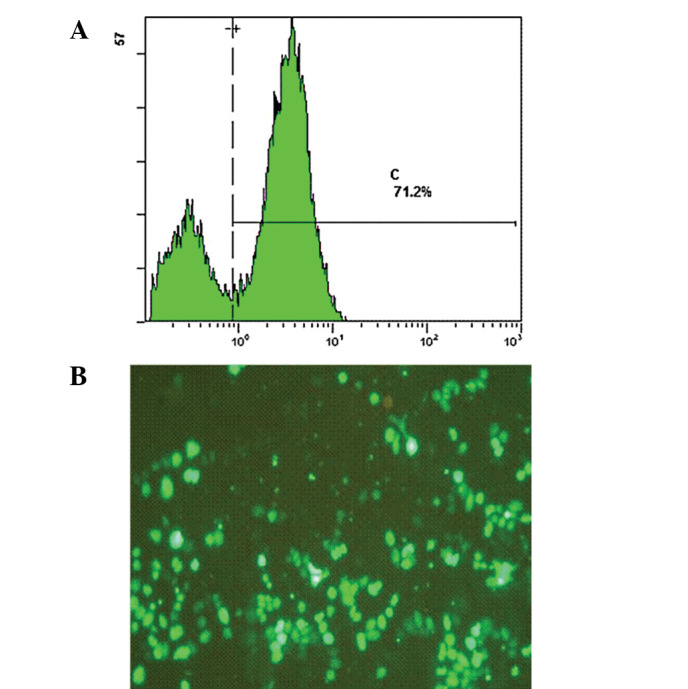
(A) Binding of EGFR/CD3 BsAb to effector cells analyzed by flow cytometry and (B) under fluorescene microscope. (FITC staining; magnification, ×100) The results showed that the binding rate of the EGFR/CD3 BsAb to CIK cells was 71.2%. EGFR, epidermal growth factor receptor; BsAb, bispecific antibody.

**Table I:** Tumor inhibition rate of each group (mean ± SD).

Group	Tumor mass (mg)	Tumor inhibition rate (%)
A	422.4±37.7^abc^	69.8±7.8^abc^
B	947.9±86.6^a^	32.2±5.1
C	724.3±61.3^ab^	48.2±6.5^ab^
D	731.9±40.8^ab^	47.6±5.5^ab^
E	1357.6±114.2	2.8±1.3
F	1012.3±91.1^a^	27.6±6.3^a^
G	989.4±87.5^a^	29.2±7.6^a^
H	1397.4±131.6	0

a P<0.05 vs. group H;

b P<0.05 vs. group B;

c P<0.05 vs. group C. Group A, EGFR/CD3 BsAb (1 mg) + CIK cells (1×10^9^/ml); group B, CD3 McAb (1 mg) + CIK cells (1×10^9^/ml); group C, EGFR McAb (1 mg) + CIK cells (1×10^9^/ml); group D, CD3 McAb (1 mg) + EGFR McAb (1 mg) + CIK cells (1×10^9^/ml); group E, CD3 McAb (1 mg); group F, EGFR McAb (1 mg); group G, EGFR/CD3 BsAb (1 mg); group H: 0.9% NaCl i.v. injection alone (0.2 ml/injection). EGFR, epidermal growth factor receptor; CIK, cytokine-induced killer; BsAb, bispecific antibody; McAb, monoclonal antibody;
